# The importance of liver function assessment before cardiac surgery: A narrative review

**DOI:** 10.3389/fsurg.2022.1053019

**Published:** 2022-12-06

**Authors:** Juan C. Lopez-Delgado, Alessandro Putzu, Giovanni Landoni

**Affiliations:** ^1^ Hospital Clinic de Barcelona, Area de Vigilancia Intensiva (ICMiD), Barcelona, Spain; ^2^ IDIBELL (Institut d’Investigació Biomèdica Bellvitge; Biomedical Investigation Institute of Bellvitge), L’Hospitalet de Llobregat, Barcelona, Spain; ^3^ Division of Anesthesiology, Department of Acute Medicine, Geneva University Hospitals, Geneva, Switzerland; ^4^ Department of Anesthesia and Intensive Care, IRCCS San Raffaele Scientific Institute, Milan, Italy; ^5^ Vita-Salute San Raffaele University, Milan, Italy

**Keywords:** cardiac surgery, cardiac anesthesia, liver disease, cirrhosis, preoperative evaluation, postoperative complications, postoperative outcome

## Abstract

The demand for cardiac surgery procedures is increasing globally. Thanks to an improvement in survival driven by medical advances, patients with liver disease undergo cardiac surgery more often. Liver disease is associated with the development of heart failure, especially in patients with advanced cirrhosis. Cardiovascular risk factors can also contribute to the development of both cardiomyopathy and liver disease and heart failure itself can worsen liver function. Despite the risk that liver disease and cirrhosis represent for the perioperative management of patients who undergo cardiac surgery, liver function is often not included in common risk scores for preoperative evaluation. These patients have worse short and long-term survival when compared with other cardiac surgery populations. Preoperative evaluation of liver function, postoperative management and close postoperative follow-up are crucial for avoiding complications and improving results. In the present narrative review, we discuss the pathophysiological components related with postoperative complications and mortality in patients with liver disease who undergo cardiac surgery and provide recommendations for the perioperative management.

## Introduction

The ageing of the population and the increase in the incidence of cardiovascular risk factors have caused a high demand for cardiac surgery in the past decades (1). At the same time, improvement in the medical care of patients with advanced liver disease led to an increased eligibility of these high-risk patients for CS (2). Furthermore, longevity contributed to the increased incidence of coronary artery disease and hepatocellular carcinoma in cirrhotic patients (3). In addition, in western countries there is an expansion of non-alcoholic fatty liver disease, also known as non-alcoholic steatohepatitis, which has risk factors similar to those of cardiovascular disease and is a frequent cause of chronic liver disease (4, 5). Viral hepatitis reached epidemic rates worldwide, and some studies showed an association between coronary artery disease and chronic hepatitis C due to enhanced endothelial injury caused by chronic inflammation in patients with liver disease (6). All these factors lead towards a high prevalence of liver disease among the whole cardiac surgery population.

Cardiac surgeons are reluctant to operate patients with advanced liver disease since this is a major perioperative risk factor in cardiac surgery with associated worse outcomes. Indeed, the severity of liver dysfunction is strongly related to an increased likelihood of complications, and the staging of liver disease must be taken into consideration in order to evaluate candidates for cardiac surgery (1). Some liver scores, such as the Child-Turcotte-Pugh (CTP) and the model for end-stage liver disease (MELD) score can be used for this purpose (see variables from CTP and MELD in Table 1) (7–11). Despite the fact that liver disease has a great impact on morbidity and mortality after cardiac surgery, it is not weighed enough within the main different systems for evaluating perioperative risk (see Table 2) in the last decade, such as the Society of Thoracic Surgeons score (STS) and European System for Cardiac Operative Risk Evaluation (EuroSCORE) II (12). These scores tend to underestimate operative risk in patients with liver disease and patients with the same characteristics have similar operative mortality risk independently of the presence of liver disease. Based on our example (see Table 3), the increase in operative risk is minimal, even when liver disease is estimated in STS score, whereas the real operative risk is quite higher (see below / Table 4). Thus, the predictability of current scores are poor in certain populations and liver function should have more weight in prognosis assessment in patients with confirmed or suspected liver disease.

**Table 1:** Variables from model for end-stage liver disease (MELD) score and child-turcotte-pugh score for cirrhosis mortality (CTP) score.

Variables	MELD^b^	CTP^c^
Renal	Creatinine	
Liver	Bilirubin	
	International Normalized Ratio	
		Albumin
		Ascites
		Encephalopathy
Electrolyte (Liver-related)	Serum sodium level^a^	

^a^ The presence of hyponatremia is a complication associated with liver disease that worsens prognosis.

^b^ Adapted from Kamath PS, Wiesner RH, Malinchoc M, Kremers W, Therneau TM, Kosberg CL, D'Amico G, Dickson ER, Kim WR. A model to predict survival in patients with end-stage liver disease. Hepatology. 2001; 33: 464-70, and Kim WR, Biggins SW, Kremers WK, Wiesner RH, Kamath PS, Benson JT, Edwards E, Therneau TM. Hyponatremia and mortality among patients on the liver-transplant waiting list. N Engl J Med. 2008; 359: 1018-26.

^c^ Adapted from Child CG, Turcotte JG. Surgery and portal hypertension. In: The liver and portal hypertension. Edited by CG Child. Philadelphia: Saunders 1964:50-64.

**Table 2:** Comparison between different variables included within main systems for evaluating perioperative risk in adult cardiac surgery.

	STS score	EuroSCORE II
***Patient related factors***		
Demographics	Age	
	Gender	
	Ethnicity	
Anthropometrics	Body Surface Area	
Toxic habits	Alcohol, tobacco and illicit drugs use	
Cardiovascular	Hypertension	
	Diabetes	Diabetes on insulin
	Peripheral artery disease	
	Family history of premature CAD	
	Syncope	
Central Nervous System	Previous cerebrovascular disease	Poor mobility
	Degree of right and left carotid stenosis	>50% of right and left carotid stenosis
Liver	Presence of Liver disease	
Lung	Sleep apnea	
	Chronic Lung Disease (i.e., lung function)	Presence of Chronic Lung Disease
	Use of home oxygen	
Renal	Preoperative creatinine levels	Renal Impairment^a^
	Need of dialysis	
Immune	Presence of Immunocompromise	
Hematology	Preoperative WBC levels	
	Preoperative Platelet levels	
Other previous severe illnesses or related treatments	Endocarditis (treated or active)	Active endocarditis
	Pneumonia	
	Cancer (within previous 5 years)	
	Mediastinal radiation	
Critical preoperative state	Inotropes	Inotropes
	Previous need of resuscitation	Preoperative cardiac massage
	IABP support	IABP support
	ECMO support	
	Catheter Based Assist Device Used	
Previous treatment	ADP inhibitors	
	ACE or ARB	
	Beta blockers	
	Steroids	
	Glycoprotein IIb/IIIa	
**Operational & surgical related factors**		
Previous procedure		
Type of previous procedure (e.g., second re-op)		
Type of current procedure		Weight of the intervention
		Surgery on thoracic aorta
Number of diseased Wessels or valvular diagnosis		
Urgency of the procedure		Urgency of the procedure
**Cardiac related factors on admission previous to surgery**		
Timing of previous MI		Recent MI (i.e., MI within 90 days)
Symptoms of angina on admission		CCS class 4 (i.e., angina at rest)
Heart failure timing		
NYHA classification		NYHA classification
Presence of cardiogenic shock		
Presence & Timing of arrhythmia (e.g., AF, flutter, ventricular, sick sinus)		
Presence & Timing of heart block		
Left Ventricular Ejection Fraction		Left Ventricular function
		Pulmonary Hypertension

STS, Society of Thoracic Surgeons; EuroSCORE II, European System for Cardiac Operative Risk Evaluation; CAD, Coronary Artery Disease; MI, Myocardial Infarction; WBC, White Blood Cells; CCS, Canadian Cardiovascular Society grading of angina pectoris; NYHA, New York Heart Association; AF, Atrial Fibrillation; ADP, Adenosine diphosphate receptor; ACE, Angiotensin-converting-enzyme inhibitors; ARB, Angiotensin II receptor blockers; IABP, Intra-aortic Balloon Pump; ECMO, Extra-Corporeal Membrane Oxygenation.

^a^ Based on creatinine clearance calculated using Cockcroft-Gault formula.

**Table 3:** Example of predicted mortality with main cardiac surgery scores compared with liver scores in patients without and with liver disease.

Characteristics of the patient		Predicted mortality			
• Male, 64 years old (75 Kg, 172 cm) • Myocardial infarction one year ago • Chronic Hepatitis C • Laboratory analysis: 42% hematocrit, 8,900 leukocytes, 220,000 platelets, creatinine 82 µmol·L^−1^ • 60% Ejection Fraction * Presence of stable Liver disease (Bilirubin: 3 mg·L^−1^, Serum Sodium Level: 136 mmol·L^−1^, INR:1.2, Albumin: 36 g·L^−1^ without any other liver associated complication)		Cardiac surgery scores		Liver disease score	
		STS	Euro SCORE II	MELD^1^	CTP^2^
Type of surgery	• Three diseased vessels • Programmed Bypass surgery	0.58% (*0.95%)	0.56% (*0.56%)	6 points *6% of estimated 3-month mortality	6 points (Child A) *10% of estimated perioperative mortality
	• Severe aortic stenosis • Programmed aortic valve replacement	0.67% (*1.007%)			
	• Two diseased vessels plus moderate mitral insufficiency • Programmed combined surgery (mitral valve replacement plus Bypass surgery)	2.01% (*2.9%)	0.97% (*0.97%)		

STS, Society of Thoracic Surgeons; EuroSCORE II, European System for Cardiac Operative Risk Evaluation; MELD, Model for End-stage Liver Disease score; CTP, Child-Turcotte-Pugh Score for Cirrhosis Mortality score; INR, International Normalized Ratio; CTP, Child-Turcotte-Pugh score; Pts., number of patients.

*Mortality with liver disease.

**Table 4:** Associated in-hospital mortality of patients with liver disease who undergo cardiac surgery based on child-turcotte-pugh score (2, 8, 10, 13, 14–25)**.**

	Year	CTP A		CTP B		CTP C		Total		
		N Death	N Pts.	N Death	N Pts.	N Death	N Pts.	N Death	N Pts.	%
Klemperer et al. (14)	1998	0	8	4	5	-	-	4	13	31%
Bizouarn et al. (13)	1999	0	10	1	2	-	-	1	12	8.3%
Suman et al. (15)	2004	1	31	5	12	1	1	7	44	15.9%
Hayashida et al. (16)	2004	0	10	2	7	1	1	3	18	16.6%
Kaplan et al. (23 )	2005	0	4	3	6	-	-	3	10	30%
Ann et al. (26)	2007	1	17	4	6	1	1	6	24	25%
Filsoufi et al. (27)	2007	1	10	2	11	4	6	7	27	26%
Yamane el al. (24)	2008	0	13	1	7	0	1	1	21	4.7%
Murashita et al. (18)	2009	3	6	1	6	-	-	4	12	33%
Morisaki et al. (28)	2010	0	30	4	12	-	-	4	42	9.5%
Thielmann et al. (8)	2010	6	39	7	14	4	4	17	57	29.8%
Gundling et al. (29)	2010	2	33	7	14	-	-	9	47	19.1%
Vanhuyse et al. (19)	2012	4	22	3	10	2	2	9	34	26.4%
Arif et al. (20)	2012	15	74	14	29	3	6	32	109	29.3%
Sugimura et al. (21)	2012	0	7	1	5	0	1	1	13	7.7%
Lopez et al. (10)	2013	0	34	5	21	2	3	7	58	12%
Morimoto et al. (22)	2013	2	14	3	21	-	-	5	35	14.3%
Komoda et al. (25)	2013	1	45	7	19	-	-	8	64	12.5%
Lin et al. (17)	2014	5	30	3	20	1	5	9	55	16.3%
Total mortality		9.6% (41 / 427)		33.9% (77 / 227)		61.3% (19 / 31)		20% (137 / 685)		

CTP, Child-Turcotte-Pugh score; Pts, number of patients.

Liver dysfunction not only affects liver physiology; it also affects the normal function of other organs, and the surgery-related inflammation may further contribute to postoperative organ dysfunctions (30). A high proportion (approximately 50%) of patients with advanced liver disease could have cardiac dysfunction, the so-called cirrhotic cardiomyopathy, a clinical syndrome characterised by reduced contractile response to stress but normal to increased cardiac output and contractility at rest (31). This may contribute to the development of acute kidney injury (AKI) or enhance chronic renal insufficiency after cardiac surgery, conditions which are closely associated with short and long-term high morbidity and mortality rate (32, 33).

Preoperative evaluation may serve to identify and optimise chronic diseases related to liver disease, such as renal disease or cirrhotic cardiomyopathy. Postoperative management and close postoperative follow-up are also crucial for avoiding severity of complications and improving outcome (34).

This narrative review aims to provide an overview of the perioperative issues related to postoperative complications in patients with liver disease who undergo cardiac surgery. However, we do not review nor give specific recommendations for liver-related complications (e.g., gastrointestinal haemorrhage, ascites, spontaneous bacterial peritonitis, hepatic encephalopathy) which were recently reported elsewhere (35). In order to provide the most rigorous data, we performed a narrative review check list to show our scientific approach in the present research (see Table S1 of Supplementary Material).

## The importance of addressing the severity of liver disease before cardiac surgery

Long-term outcomes of patients with liver disease who undergo cardiac surgery are closely related to the preoperative severity of liver disease and to the incidence of complications during the perioperative period (13). We identified the following predictors of mortality after cardiac surgery in these patients: older age, increased preoperative total plasma bilirubin, increased International Normalised Ratio (INR), low preoperative serum cholinesterase levels, high central venous pressure, preoperative congestive heart failure, preoperative weight loss, preoperative and postoperative thrombocytopenia; prolonged cardiopulmonary bypass (CPB) time; and prolonged operative time (2, 8, 10, 14–25, 36, 37). Thus, both liver-related variables (i.e., preoperative) and those related to the development of the inflammatory response after cardiac surgery (i.e., intraoperative) have a higher impact on the outcome of these patients.

As we previously mention, the EuroSCORE and the STS score are both widely accepted in Europe and America, respectively, as valuable scores in cardiac surgery, but they do not consider liver function (38, 39). Liver scores such as MELD or CTP can be useful for staging liver function before cardiac surgery in patients with liver disease (7, 11). Mortality is higher in patients with liver cirrhosis in comparison with the non-cirrhotic population in all CTP score groups, especially in those classified as class B and C (8, 10). We collected and reported in Table 4 all mortality in-hospital mortality rates of patients who underwent cardiac surgery with liver disease based on CTP score (2, 8, 10, 13–25). The postoperative long-term mortality rates after hospital discharge are even also higher for cirrhotic patients undergoing cardiac surgery, ranging from 40% to 70% at about six years follow-up (34). A high MELD score (see Table 1) with a cut-off ranging from 13 to 18 is associated with a worse prognosis after cardiac surgery in patients with liver disease (8–10). MELD has the advantage of being more objective than the CTP score because it has measurable variables, and it reflects dynamic changes in liver function that could be corrected by the improvement of cardiac insufficiency (7, 40). Despite the fact that MELD was a better predictor of mortality in some studies which compare it to CTP, CTP score remains a reliable tool for staging liver disease before cardiac surgery (11, 15, 25). Indeed, the MELD score may overestimate liver disease because it can be influenced by worsening of cardiac insufficiency related renal function or because INR may be affected by the anticoagulation therapies that are used to treat cardiac-related problems such as atrial fibrillation (40, 41).

In the immediate postoperative period, ICU mortality risk scores, such as the simplified acute physiology score (SAPS) III, may be used in this specific population since they include liver-related variables (e.g., albumin, platelets, renal function) (10).

The presence of chronic hepatitis, especially hepatitis C, must be considered for preoperative evaluation because it is associated with high postoperative haemorrhage and surgical revision for bleeding (even in the presence of minor changes in INR), infection and postoperative cardiac complications (42–44). Therefore, viral load should be assessed before cardiac surgery.

As a general rule, cardiac surgery may be performed with higher operative risk in patients without advanced liver disease (i.e., Child A), but mortality is worsened with both higher CTP class and MELD score. Patients staged in CTP class B and MELD between 9 and 14 should be carefully evaluated before cardiac surgery, whereas in patients with CTP class C and MELD ≥15, cardiac surgery should be contraindicated. The weight of both liver scores in establishing indication for cardiac surgery may be considered equivalent and complementary. Similar to other major surgeries in high-risk patients, the ultimate decision of performing cardiac surgery should also consider medical past history (e.g., previous liver-related complications), the individual evaluation of the patient in terms of surgical risk and the potential benefit, especially in those patients in whom clinicians have doubts about performing or not cardiac surgery (e.g., CTP class B and MELD 15). For example, the combined presence of severe portal hypertension with liver-related complications, such as several episodes of variceal bleeding, and CTP class B, may imply a high-risk for mortality and a contraindication for surgery. However, indication of cardiac surgery should also balance the urgency in correcting the surgical problem together with the risk that cardiac surgery entails *per se*. If liver function cannot be optimized before surgery, this may represent poor liver functional reserve and cardiac surgery may be contraindicated. We suggest the use of both liver scores in conjunction with cardiac scores for the preoperative evaluation of patients with liver disease before cardiac surgery.

Liver function may be optimised in some patients, even though liver transplantation, in order to shift the stage of liver disease towards a less severe staging and ultimately give the opportunity to perform cardiac surgery. In Table 5, we show a summary of recommendations for the improvement of perioperative management in patients who undergo cardiac surgery with liver disease. Despite those recommendations are based in small studies, they are supported by the following pathophysiological considerations.

**Table 5:** Specific recommendations for the perioperative management of patients with liver disease who undergo cardiac surgery.

	Preoperative	Intraoperative	Postoperative
Liver function	Calculate MELD and CTP scores (7, 11)	Consider (45–52): - Perfusion flow rates ≥ 2.3 L/min and pulsatile flow - Revascularisation surgery performed by off-pump techniques - Aortic valve replacement performed by means of transcatheter techniques - Albumin as priming solution - Minimise CPB and operation time as much as possible - Avoid urgent procedures	Close monitoring of liver function & optimise treatment for liver disease (e.g., lactulose and avoid diuretics when possible to avoid liver encephalopathy) (30, 34, 35)
	Avoid cardiac surgery in CTP C and MELD ≥15, and evaluate carefully in CTP A, CTP B and MELD <15 (2, 8, 10, 13–25)		
	Evaluate hepatic reserve (i.e., Evaluate if there is any improvement of INR afer the administration of Vitamin K) (53)		
	Evaluate previous liver-related complications (e.g., ascites, liver encephalopathy, gastric bleeding) and optimise treatment for liver disease (30, 35)		
Coagulation	Evaluate and correct coagulopathy throughout thromboelastography and platelet function at least 24 h before surgery (23, 54–56)	Guide transfusion therapy by means of thromboelastography (54–56)	Close monitoring and small increase in dosage when anticoagulant therapy is given (57, 58)
		Avoid hypothermia, haemodilution and massive transfusion (59)	
Cardiovascular function	Stress echocardiography with dobutamine to evaluate cardiac reserve and function (31, 32)	Avoid positive fluid balance as much as possible (10, 34, 60)	Avoid positive fluid balance & optimise haemodynamic status (10, 34, 60)
	Consider *β*-blocker administration (if not contraindicated) (61, 62)		
	Evaluate the degree of cardiac insufficiency and prognosis by means of BNP (63)		
	Optimise preoperative volume status (10)		
Renal Function	Avoid drugs associated with nephrotoxicity before CS (33, 64)		
	Optimise preoperative haemodynamic status / cardiac function (33, 64)		
Immune response (65)	Screening for multi-resistant pathogens	Avoid transfusions	Early diagnosis of infection and administration of antibiotics
			Prevention of nosocomial infections
Nutritional status (66–69)	Avoid cardiac surgery in patients with albumin <30–35 g·L^−1^ and optimize nutritional status	Avoid long fasting times	Early nutritional support with oral, enteral or parenteral nutrition
	Preoperative nutritional support (involve nutritionists & nurses)		
Pulmonary function (70–72)	Preoperative medical history of respiratory failure	Avoid positive fluid balance as much as possible	Early physiotherapy
	Screen for pulmonary hypertension when preoperative right heart failure is present and make a diagnosis		Consider the use of non-invasive mechanical ventilation to prevent atelectasis
	Avoid surgery in patients with hepatopulmonary syndrome		Apply specific treatments for pulmonary hypertension when necessary

CS, Cardiac surgery; LD, Liver disease; CTP, Child-Turcotte-Pugh; MELD, Model for end-stage liver disease score; INR, International Normalised Ratio; BNP, Brain natriuretic peptide.

## Pathophysiological considerations of liver disease in cardiac surgery

The progression of liver disease involves the development of portal hypertension, which can lead to ascites, spontaneous bacterial peritonitis, variceal bleeding, and hepatic encephalopathy (35). Liver-related complications may be frequent postoperatively, ranging from 8% to 33% depending on the severity of liver disease (2, 8, 10, 13, 14–25). Variceal bleeding and ascites are the most common complications, especially in the presence of previous portal hypertension or medical past history of variceal bleeding (8, 10). Hepatic encephalopathy is poorly described in these patients, but it may happen when patient suffer from cirrhosis (14). Patients with liver disease are at higher risk of liver-related complications but advanced liver disease (i.e., cirrhosis) further predisposes to higher incidence non-liver-related complications after cardiac surgery (27, 30).

### Coagulation

Bleeding is one of the main concerns during cardiac surgery and the liver is the main source of coagulation proteins. Advanced liver disease is associated with a decrease in both pro- and anti-coagulants factors together with thrombocytopenia, and severe bleeding occurs in up to 30% of patients with liver disease after cardiac surgery (30). Hypothermia, haemodilution and hypoperfusion produced by CPB and fluid administration aggravate coagulopathy produced by liver disease during cardiac surgery (59). Factors associated with liver disease, such as poor nutrition, hypoalbuminaemia and renal failure may contribute to platelet dysfunction (73). Thus, excessive mediastinal bleeding is a frequent complication that leads to increased transfusion requirements and reoperation for bleeding (23).

The INR is used to assess prognosis in the MELD score, bleeding risk, and to guide treatment of some type of coagulation disturbances in clinical practice. The lack of improvement of INR with the preoperative administration of vitamin K may reflect a poor liver reserve, so it may be used for preoperative evaluation (30). Even though INR provides a good measure of liver function, it only measures the activity of some of the coagulant factors (53). Thromboelastography (TEG) is a tool that might provide better assessment of the degree of coagulopathy, which would enable immediate (i.e., from 30 min to 1 h) and goal-oriented transfusion therapy (54). TEG provides in which component(s) of the coagulation process (i.e., fibrinogen, platelets, intrinsic or extrinsic coagulation pathway) is the coagulative problem (if any) (54). This is especially useful in cardiac surgery because TEG enables a more individualized transfusion therapy, it could optimize preoperative and even perioperative coagulation status, and it may also improve outcomes (i.e., Length of stay, bleeding rate, and mortality) (54–56).

Preventive strategies aiming to optimise coagulopathy after cardiac surgery are also necessary (53–56). Oral anticoagulant use following cardiac surgery may entail higher risk in patients with liver disease and may potentially cause fatal bleeding complications (e.g., intracranial haemorrhage) and anticoagulant-induced liver failure (57, 58). Thus, the use of biological cardiac valves and grafts when possible, and a close bleeding and coagulation monitoring when oral anticoagulants are needed may be suggested. Despite there is an inherent risk of bleeding, deep vein thrombosis prophylaxis should be performed during perioperative period, especially in those patients with liver disease prone to suffer perioperative thrombotic complications (e.g., splanchnic or portal vein thrombosis, liver tumors, venous thromboembolism and Budd Chiari syndrome) (30, 55, 74).

### Immune response

The presence of an innate and adaptive immune system dysfunction which is defined as cirrhosis-associated immune dysfunction syndrome, predisposes patients with liver disease to systemic infections. Depression and stimulation of the immune system coexist, resulting in a high susceptibility to acute inflammatory and infective processes (75). There is also a shift towards persistent inflammation leading to liver fibrosis progression and development of different complications, such as portal hypertension and hepatic encephalopathy (76). Sepsis occurs in approximately 60% of patients and is the main cause of postoperative mortality after cardiac surgery through multi-system organ failure (10, 30). Gastrointestinal bleeding, low serum albumin, poor nutritional status and higher re-exploration rates are associated with increased rates of infection in liver disease (65). Blood transfusions after cardiac surgery are associated with an increased risk of infection at multiple sites, suggesting a system-wide immune dysfunction response (77). Sepsis is an important risk factor for mortality following cardiac surgery because it can produce a sepsis-induced cardiac dysfunction, which can ultimately enhance postoperative low cardiac output syndrome and limit fluid responsiveness (78). Screening for multi-resistant pathogens, early diagnosis of infection, early administration of antibiotics, and a careful prevention of nosocomial infections are among the strategies that may be recommended during the perioperative course of cardiac surgery.

### Nutritional status

The correct functioning of the immune and metabolic response systems are both interdependent (66). Different factors lead to an insufficient nutritional reserve in patients with liver disease (i.e., poor nutritional intake, inappropriate absorption due to gastrointestinal dysfunction, persistent inflammation), and in consequence the response to surgical stress is metabolically inefficient (67). Preoperative serum albumin level can be used to assess nutritional status and underlying disease before cardiac surgery, with levels of serum albumin <25 g·L^−1^ being independently associated with an increased risk of reoperation for bleeding (66, 68). Lower levels of hypoalbuminaemia are also associated with increased risk of infection after cardiac surgery, and guidelines do not recommend surgery in patients with albumin <30–35 g·L^−1^ (68, 69). The lack of response (i.e., lack of improvement in albumin or prealbumin levels) to preoperative nutritional support may serve as a surrogate marker of poor hepatic reserve before cardiac surgery (69).

### Cardiovascular function

Patients with liver disease suffer from cardiac dysfunction and poor cardiac reserve (32). Liver disease coexists with peripheral arterial vasodilatation due to reduced arterial compliance and activation of sodium and water retentive pathways that produce expansion and redistribution of blood volume in the splanchnic bed. Consequently, there is a resting hyperdynamic hemodynamic state with increased cardiac output in response to these alterations. These alterations progress in parallel with liver disease, leading to cardiac failure and cirrhotic cardiomyopathy. These are characterised by diastolic dysfunction with poorer inotropic and chronotropic response (31, 32). The assessment of preoperative cardiac function by means of echocardiography, even with dobutamine to evaluate stress responsiveness, could play a role in the preoperative evaluation and postoperative management in patients with liver disease undergoing cardiac surgery. A poor cardiac tolerance to stress echocardiography may indicate poor prognosis and higher probability of cardiac complications (e.g., low cardiac output syndrome) after cardiac surgery (31, 32). Brain natriuretic peptide, a marker of cardiac dysfunction useful in diagnosing heart failure, is correlated with the CTP score, occurrence of liver-related complications and 1-year mortality in patients with liver disease (63). Thus, stress echocardiography and brain natriuretic peptide may be used together to evaluate cardiac function preoperatively. In each cardiac surgery candidates, elevated preoperative or postoperative cardiac troponin could be associated with higher postoperative morbidity and mortality, especially in the case of urgent cardiac surgery due to acute myocardial ischaemia (61, 79).

Cardiovascular disease is also a common cause of morbidity and mortality in patients with liver disease (31). The histologic severity of liver injury and inflammation is associated with an increased cardiovascular risk and an atherogenic lipid profile (62). If we exclude recurrent disease, graft loss resulting from technical complications, and malignancies, cardiac complications are among the most common complications after liver transplantation (34). Furthermore, liver allograft recipients have a greater risk of cardiovascular deaths and ischemic events than an age- and sex-matched population (80). A history of coronary artery disease, prior stroke, increased interventricular septal thickness, and postoperative sepsis are markers of adverse perioperative cardiac outcomes after liver transplantation, whereas use of perioperative beta-blockers was significantly protective (81). The same may be applied to patients with advanced liver disease undergoing CS in the absence of contraindications for beta-blocker administration, especially with the presence of diastolic dysfunction (32, 34).

Moderate to advanced heart failure can produce liver disease secondary to liver congestion (i.e., cardiac or congestive hepatopathy) (60). Advanced congestion, low cardiac output that causes hypoperfusion, and right-heart failure caused by severe tricuspid regurgitation or pulmonary hypertension (i.e., pulmonary artery systolic pressure >45 mmHg) are among its causes (82). Cardiac hepatopathy is usually partially or completely reversed when cardiac problems are corrected.

Haemodynamic postoperative management is key for cardiac complications, suggesting the need for a careful management of preload and fluid balance, which ultimately may influence postoperative renal and respiratory function (83). It is not surprising that higher central venous pressure is associated with higher short-term mortality in these patients (10). Addressing cardiovascular dysfunction after cardiac surgery is crucial, even during the follow-up after hospital discharge, since liver and heart failure are among the main causes of hospital readmission in patients who underwent cardiac surgery with liver disease (34).

### Renal function

Liver disease is related with renal dysfunction, the so called hepatorenal syndrome. This is associated with tissue and microcirculatory dysfunction in the heart and in the peripheral vascular bed (64). Oliguria could be a clinical sign of AKI, a common postoperative complication associated with higher short- and long-term mortality in cardiac surgery (33). Renal failure could lead to positive fluid balance, resulting in vital organs oedema and failure (83). Low urine output in the first 24 h following cardiac surgery is a valuable predictor of long-term outcome in patients with liver disease undergoing cardiac surgery (36). Patients with liver disease suffer from renal dysfunction more frequently after cardiac surgery than patients without liver disease (70). AKI has an incidence as high as 80% in cirrhotic patients after cardiac surgery, with up to 40% of these patients needing renal replacement therapies (30). We suggest that optimising haemodynamic status, avoiding nephrotoxic drugs that may enhance or cause renal failure, and avoiding positive fluid balance as much as possible are among the strategies to improve perioperative renal function (33, 71).

### Pulmonary function

Ascites and fluid overload may cause or worsen pulmonary function due to atelectasis and heart failure. In patients with liver disease, severe pulmonary complications (i.e., those that cause respiratory insufficiency) occurs in up to 30% of cases (30).

Typical pulmonary complications in advanced liver disease include hepatopulmonary syndrome, portopulmonary hypertension and hepatic hydrothorax (72). Whereas hepatopulmonary syndrome and portopulmonary hypertension represent pulmonary vascular diseases, the development of hepatic hydrothorax is associated with the presence of ascites and phrenic nerve lesions. For severe hepatopulmonary syndrome and refractory hepatic hydrothorax, liver transplantation is the treatment of choice (45). Specific medical therapy is indicated in severe portopulmonary hypertension (e.g., intravenous prostanoids, oral endothelin receptor antagonists and phosphodiesterase-5 inhibitors) (45, 46, 72). All these complications need to be screened, especially in patients with a history of respiratory failure and moderate or advanced liver disease. Avoiding positive fluid balance and optimising right heart function may be helpful to reduce respiratory complications. Early physiotherapy should also be performed in order to avoid atelectasis (46).

## Pathophysiological considerations for intraoperative management

Cardiac surgery involves a systemic inflammatory response mostly due to CPB, transfusions and surgical trauma. These together lead to poor hepato-splanchnic perfusion that can cause intestinal mucosal injury, therefore predisposing to endotoxemia and proinflammatory cytokine release (47). Extracorporeal CPB circuit stimulates inflammation by activation of the intrinsic coagulation pathway, worsening the coagulopathy of liver disease (48). Furthermore, the perioperative risks associated with all major cardiac surgery procedures are enhanced in the presence of liver disease due to the higher immunologic and metabolic demands that the surgical procedure imposes to the liver (2, 8, 10, 13, 14–25). The non-physiologic and non-pulsatile CPB flow could lead to ischaemia-reperfusion liver injury in association to low cardiac output episodes. A decrease in liver perfusion by about 20% and in arterial blood flow by 20%–45% through vasoconstriction during CPB could result in an imbalance in oxygen supply (49).

Perioperative strategies should focus on minimising CPB duration (<100 min) and minimising transfusion requirements. High perfusion flow rates (≥ 2.3 L/min) might have a beneficial effect on liver function and overall organ perfusion (50–52). As a consequence, there is a potential benefit when revascularisation surgery is performed off-pump and when aortic valve replacement is performed by means of transcatheter techniques (84). Albumin could be used as priming solution for CPB in order to improve bleeding-related complications (85). Emergent procedures should be avoided in order to optimise patients with liver disease as much as possible. This is especially true in patients with liver disease and infective endocarditis, who are prone to an increased risk of bacteraemia associated with invasive procedures and immune dysfunction (86).

## Conclusions

Liver disease remains a major risk factor during the perioperative course of cardiac surgery. The pathophysiological characteristics of severe liver disease predispose to increased rates of complications and higher short- and long-term mortality when cardiac surgery is performed in these patients. All the different pathophysiological considerations of liver disease should be carefully evaluated prior to the performance of cardiac surgery in patients with liver disease. Liver scores (i.e., MELD and CTP) may be useful for the assessment of perioperative risk in those patients.

Optimize perfusion (e.g., Perfusion flow rates ≥2.3 L/min) and minimize surgical-related inflammation (e.g., minimise CPB and operation time as much as possible) are both key for avoiding perioperative liver dysfunction. The choice of less invasive and aggressive surgical techniques (e.g., transcatheter techniques) and the minimisation of surgical injury (e.g., off-pump revascularisation) may have a beneficial effect on the outcome of these patients. Close monitorization of coagulation and platelet function (e.g., TEG), and avoid hypothermia as much as possible, are both important to avoid bleeding and optimize transfusion policy. Fluid management avoiding positive fluid balance as much as possible may be useful to improve perioperative cardiovascular, renal, and even pulmonary and coagulopathy (i.e., hemodilution) status of these patients. All these considerations, together with a close attention for the prevention of nosocomial infections, preoperative optimization of physical status and early postoperative physiotherapy, as well as a plan to improve preoperative nutritional status and an early postoperative nutrition therapy, may be helpful to improve perioperative outcomes and prevent potential complications (see detailed recommendations on Table 5). Even though evidence comes mainly from small studies, some recommendations could be given for their perioperative management, even to improve preoperatively liver function.

## References

[1] Benjamin EJ Virani SS Callaway CW Chamberlain AM Chang AR Cheng S Heart disease and stroke statistics-2018 update: a report from the American heart association. Circulation. (2018) 137:e67–e492. 10.1161/CIR.0000000000000558 29386200

[2] Araujo L Dombrovskiy V Kamran W Lemaire A Chiricolo A Lee LY The effect of preoperative liver dysfunction on cardiac surgery outcomes. J Cardiothorac Surg. (2017) 12:73. 10.1186/s13019-017-0636-y 28865456 PMC5581433

[3] Shaheen AA Kaplan GG Hubbard JN Myers RP. Morbidity and mortality following coronary artery bypass graft surgery in patients with cirrhosis: a population-based study. Liver Int. (2009) 29:1141–51. 10.1111/j.1478-3231.2009.02058.x 19515218

[4] Vernon G Baranova A Younossi ZM. Systematic review: the epidemiology and natural history of non-alcoholic fatty liver disease and non-alcoholic steatohepatitis in adults. Aliment Pharmacol Ther. (2011) 34:274–85. 10.1111/j.1365-2036.2011.04724.x 21623852

[5] Estes C Anstee QM Arias-Loste MT Bantel H Bellentani S Caballeria J Modeling NAFLD disease burden in China, France, Germany, Italy, Japan, Spain, United Kingdom, and United States for the period 2016-2030. J Hepatol. (2018) 69(4):896–904. 10.1016/j.jhep.2018.05.036 29886156

[6] Adinolfi LE Zampino R Restivo L Lonardo A Guerrera B Marrone A Chronic hepatitis C virus infection and atherosclerosis: clinical impact and mechanisms. World J Gastroenterol. (2014) 20:3410–7. 10.3748/wjg.v20.i13.3410 24707124 PMC3974508

[7] Murata M Kato TS Kuwaki K Yamamoto T Dohi S Amano A. Preoperative hepatic dysfunction could predict postoperative mortality and morbidity in patients undergoing cardiac surgery: utilization of the MELD scoring system. Int J Cardiol. (2016) 203:682–9. 10.1016/j.ijcard.2015.10.181 26583843

[8] Thielmann M Mechmet A Neuhäuser M Wendt D Tossios P Canbay A Risk prediction and outcomes in patients with liver cirrhosis undergoing open-heart surgery. Eur J Cardiothorac Surg. (2010) 38:592–9. 10.1016/j.ejcts.2010.02.042 20413316

[9] Modi A Vohra HA Barlow CW. Do patients with liver cirrhosis undergoing cardiac surgery have acceptable outcomes? Interact CardioVasc Thorac Surg. (2010) 11:630–4. 10.1510/icvts.2010.241190 20739405

[10] Lopez-Delgado JC Esteve F Javierre C Perez X Torrado H Carrio ML Short-term independent mortality risk factors in patients with cirrhosis undergoing cardiac surgery. Interact CardioVasc Thorac Surg. (2013) 16:332–8. 10.1093/icvts/ivs501 23243034 PMC3568816

[11] Jacob KA Hjortnaes J Kranenburg G de Heer F Kluin J. Mortality after cardiac surgery in patients with liver cirrhosis classified by the Child-Pugh score. Interact Cardiovasc Thorac Surg. (2015) 20:520–30. 10.1093/icvts/ivu438 25612743

[12] Ranucci M Guarracino F Castelvecchio S Baldassarri R Covello RD Landoni G, ACEF Score Research Group. Surgical and transcatheter aortic valve procedures. The limits of risk scores. Interact Cardiovasc Thorac Surg. (2010) 11(2):138–41. 10.1510/icvts.2010.236141 20484408

[13] Bizouarn P Ausseur A Desseigne P Le Teurnier Y Nougarede B Train M Early and late outcome after elective cardiac surgery in patients with cirrhosis. Ann Thorac Surg. (1999) 67:1334–8. 10.1016/S0003-4975(99)00226-X 10355407

[14] Klemperer JD Ko W Krieger KH Connolly M Rosengart TK Altorki NK Cardiac operations in patients with cirrhosis. Ann Thorac Surg. (1998) 65:85–7. 10.1016/S0003-4975(97)00931-4 9456100

[15] Suman A Barnes DS Zein NN Levinthal GN Connor JT Carey WD. Predicting outcome after cardiac surgery in patients with cirrhosis: a comparison of Child-Pugh and MELD scores. Clin Gastroenterol Hepatol. (2004) 2:719–23. 10.1016/S1542-3565(04)00296-4 15290666

[16] Hayashida N Shoujima T Teshima H Yokokura Y Takagi K Tomoeda H Clinical outcome after cardiac operations in patients with cirrhosis. Ann Thorac Surg. (2004) 77:500–5. 10.1016/j.athoracsur.2003.06.021 14759426

[17] Lin CH Hsu RB. Cardiac surgery in patients with liver cirrhosis: risk factors for predicting mortality. World J Gastroenterol. (2014) 20:12608–14. 10.3748/wjg.v20.i35.12608 25253965 PMC4168098

[18] Murashita T Komiya T Tamura N Sakaguchi G Kobayashi T Furukawa T Preoperative evaluation of patients with liver cirrhosis undergoing open heart surgery. Gen Thorac Cardiovasc Surg. (2009) 57:293–7. 10.1007/s11748-008-0374-0 19533274

[19] Vanhuyse F Maureira P Portocarrero E Laurent N Lekehal M Carteaux JP Cardiac surgery in cirrhotic patients: results and evaluation of risk factors. Eur J Cardiothorac Surg. (2012) 42:293–9. 10.1093/ejcts/ezr320 22290926

[20] Arif R Seppelt P Schwill S Kojic D Ghodsizad A Ruhparwar A Predictive risk factors for patients with cirrhosis undergoing heart surgery. Ann Thorac Surg. (2012) 94:1947–52. 10.1016/j.athoracsur.2012.06.057 22921237

[21] Sugimura Y Toyama M Katoh M Kato Y Hisamoto K. Analysis of open heart surgery in patients with liver cirrhosis. Asian Cardiovasc Thorac Ann. (2012) 20:263–8. 10.1177/0218492311435339 22718713

[22] Morimoto N Okada K Okita Y. The model for end-stage liver disease (MELD) predicts early and late outcomes of cardiovascular operations in patients with liver cirrhosis. Ann Thorac Surg. (2013) 96:1672–8. 10.1016/j.athoracsur.2013.06.007 23987897

[23] Kaplan M Cimen S Kut MS Demirtas MM. Cardiac operations for patients with chronic liver disease. Heart Surg Forum. (2002) 5:60–5.. 11937465

[24] Yamane K Izumi K Yamachika S Hashizume K Tanigawa K Miura T Operative outcome of cardiac surgery in patients with liver cirrhosis. Acta Med Nagasaki. (2008) 53:15–21. 10.11343/amn.53.15

[25] Komoda T Frumkin A Knosalla C Hetzer R. Child-Pugh score predicts survival after radical pericardiectomy for constrictive pericarditis. Ann Thorac Surg. (2013) 96:1679–85. 10.1016/j.athoracsur.2013.06.016 23998414

[26] An Y Xiao YB Zhong QJ. Open-heart surgery in patients with liver cirrhosis: indications, risk factors, and clinical outcomes. Eur Surg Res. (2007) 39:67–74. 10.1159/000099145 17283429

[27] Filsoufi F Salzberg SP Rahmanian PB Schiano TD Elsiesy H Squire A Early and late outcome of cardiac surgery in patients with liver cirrhosis. Liver Transpl. (2007) 13:990–5. 10.1002/lt.21075 17427174

[28] Morisaki A Hosono M Sasaki Y Kubo S Hirai H Suehiro S Risk factor analysis in patients with liver cirrhosis undergoing cardiovascular operations. Ann Thorac Surg. (2010) 89:811–7. 10.1016/j.athoracsur.2009.12.021 20172135

[29] Gundling F Seidl H Gansera L Schuster T Hoffmann E Kemkes BM Early and late outcomes of cardiac operations in patients with cirrhosis: a retrospective survival-rate analysis of 47 patients over 8 years. Eur J Gastroenterol Hepatol. (2010) 22:1466–73. 10.1097/MEG.0b013e32834059b6 21346421

[30] Lopez-Delgado JC Esteve F Javierre C Ventura JL Mañez R Farrero E Influence of cirrhosis in cardiac surgery outcomes. World J Hepatol. (2015) 7:753–60. 10.4254/wjh.v7.i5.753 25914775 PMC4404380

[31] Møller S Danielsen KV Wiese S Hove JD Bendtsen F. An update on cirrhotic cardiomyopathy. Expert Rev Gastroenterol Hepatol. (2019) 13:497–505. 10.1080/17474124.2019.1587293 30802157

[32] Theocharidou E Krag A Bendtsen F Møller S Burroughs AK. Cardiac dysfunction in cirrhosis - does adrenal function play a role? A hypothesis. Liver Int. (2012) 32:1327–32. 10.1111/j.1478-3231.2011.02751.x 22292920

[33] Lopez-Delgado JC Esteve F Torrado H Rodríguez-Castro D Carrio ML Farrero E Influence of acute kidney injury on short- and long-term outcomes in patients undergoing cardiac surgery: risk factors and prognostic value of a modified RIFLE classification. Crit Care. (2013) 17:R293. 10.1186/cc13159 24330769 PMC4056889

[34] Chou AH Chen TH Chen CY Chen SW Lee CW Liao CH Long-Term outcome of cardiac surgery in 1,040 liver cirrhosis patient - nationwide population-based cohort study. Circ J. (2017) 81:476–84. 10.1253/circj.CJ-16-0849 28163280

[35] Nusrat S Khan MS Fazili J Madhoun MF. Cirrhosis and its complications: evidence-based treatment. World J Gastroenterol. (2014) 20:5442–60. 10.3748/wjg.v20.i18.5442 24833875 PMC4017060

[36] Lopez-Delgado JC Esteve F Javierre C Torrado H Carrio ML Rodríguez-Castro D Predictors of long-term mortality in patients with cirrhosis undergoing cardiac surgery. J Cardiovasc Surg (Torino). (2014) 56:647–54. 24670881

[37] Steffen RJ Bakaeen FG Vargo PR Kindzelski BA Johnston DR Roselli EE Impact of cirrhosis in patients who underwent surgical aortic valve replacement. Am J Cardiol. (2017) 120:648–54. 10.1016/j.amjcard.2017.05.034 28693742

[38] Hawkins RB Young BAC Mehaffey JH Speir AM Quader MA Rich JB Investigators for the Virginia cardiac services quality initiative. Model for End stage liver disease score independently predicts mortality in cardiac surgery. Ann Thorac Surg. (2019) 107(6):1713–9. 10.1016/j.athoracsur.2018.12.011 30639362 PMC6541453

[39] Ad N Holmes SD Patel J Pritchard G Shuman DJ Halpin L. Comparison of EuroSCORE II, original EuroSCORE, and the society of thoracic surgeons risk score in cardiac surgery patients. Ann Thorac Surg. (2016) 102:573–9. 10.1016/j.athoracsur.2016.01.105 27112651

[40] Heuman DM Mihas AA Habib A Gilles HS Stravitz RT Sanyal AJ MELD-XI: a rational approach to “sickest first” liver transplantation in cirrhotic patients requiring anticoagulant therapy. Liver Transpl. (2007) 13:30–7. 10.1002/lt.20906 17154400

[41] Ha NB Regal RE. Anticoagulation in patients with cirrhosis: caught between a rock-liver and a hard place. Ann Pharmacother. (2016) 50:402–9. 10.1177/1060028016631760 26861989

[42] Baran C Cakici M Ozcinar E Durdu S Inan B Sirlak M Clinical results of cardiac surgery in patients with chronic hepatitis C and their role in risk models: a case-control study. Thorac Cardiovasc Surg. (2018) 66:328–32. 10.1055/s-0037-1599058 28282660

[43] Emerson D Endicott K Amdur R Trachiotis G. Cardiac surgery in patients chronically infected with hepatitis C virus: long-term outcomes and comparison to historical controls and human immunodeficiency virus infection. J Cardiovasc Surg (Torino). (2016) 57:598–605. 25318844

[44] Hsieh WC Chen PC George G Tinica G Corciova FC. Prevalence of post-operative morbidity risk factors following cardiac surgery in patients with chronic viral hepatitis: a retrospective study. Eur Rev Med Pharmacol Sci. (2015) 19:2575–82. 26221885

[45] Huffmyer JL Nemergut EC. Respiratory dysfunction and pulmonary disease in cirrhosis and other hepatic disorders. Respir Care. (2007) 52:1030–6. 17650360

[46] Surani SR Mendez Y Anjum H Varon J. Pulmonary complications of hepatic diseases. World J Gastroenterol. (2016) 22:6008–15. 10.3748/wjg.v22.i26.6008 27468192 PMC4948262

[47] Corral-Velez V Lopez-Delgado JC Betancur-Zambrano NL Lopez-Suñe N Rojas-Lora M Torrado H The inflammatory response in cardiac surgery: an overview of the pathophysiology and clinical implications. Inflamm Allergy Drug Targets. (2015) 13:367–70. 10.2174/1871528114666150529120801 26021321

[48] Paparella D Brister SJ Buchanan MR. Coagulation disorders of cardiopulmonary bypass: a review. Intensive Care Med. (2004) 30:1873–81. 10.1007/s00134-004-2388-0 15278267

[49] Di Tomasso N Monaco F Landoni G. Hepatic and renal effects of cardiopulmonary bypass. Best Pract Res Clin Anaesthesiol. (2015) 29:151–61. 10.1016/j.bpa.2015.04.001 26060027

[50] Fromes Y Gaillard D Ponzio O Chauffert M Gerhardt MF Deleuze P Reduction of the inflammatory response following coronary bypass grafting with total minimal extracorporeal circulation. Eur J Cardiothorac Surg. (2002) 22:527–33. 10.1016/s1010-7940(02)00372-x 12297167

[51] Liu Y Tao L Wang X Cui H Chen X Ji B. Beneficial effects of using a minimal extracorporeal circulation system during coronary artery bypass grafting. Perfusion. (2012) 27:83–9. 10.1177/0267659111424636 21987667

[52] Mathie RT. Hepatic blood flow during cardiopulmonary bypass. Crit Care Med. (1993) 21:S72–6. 10.1097/00003246-199302001-00013 8428501

[53] Vang ML Hvas AM Ravn HB. Urgent reversal of vitamin K antagonist therapy. Acta Anaesthesiol Scand. (2011) 55:507–16. 10.1111/j.1399-6576.2011.02414.x 21418150

[54] Dias JD Sauaia A Achneck HE Hartmann J Moore EE. Thromboelastography-guided therapy improves patient blood management and certain clinical outcomes in elective cardiac and liver surgery and emergency resuscitation: a systematic review and analysis. J Thromb Haemost. (2019) 17:984–94. 10.1111/jth.14447 30947389 PMC6852204

[55] De Pietri L Bianchini M Rompianesi G Bertellini E Begliomini B. Thromboelastographic reference ranges for a cirrhotic patient population undergoing liver transplantation. World J Transplant. (2016) 6:583–93. 10.5500/wjt.v6.i3.583 27683637 PMC5036128

[56] Redfern RE Fleming K March RL Bobulski N Kuehne M Chen JT Thrombelastography-Directed transfusion in cardiac surgery: impact on postoperative outcomes. Ann Thorac Surg. (2019) 107:1313–8. 10.1016/j.athoracsur.2019.01.018 30768933

[57] Lee SR Lee HJ Choi EK Han KD Jung JH Cha MJ Direct oral anticoagulants in patients with atrial fibrillation and liver disease. J Am Coll Cardiol. (2019) 73:3295–308. 10.1016/j.jacc.2019.04.052 31248551

[58] Efird LM Mishkin DS Berlowitz DR Ash AS Hylek EM Ozonoff A Stratifying the risks of oral anticoagulation in patients with liver disease. Circ Cardiovasc Qual Outcomes. (2014) 7:461–7. 10.1161/CIRCOUTCOMES.113.000817 24823958

[59] Nogami K. The utility of thromboelastography in inherited and acquired bleeding disorders. Br J Haematol. (2016) 174:503–14. 10.1111/bjh.14148 27264484

[60] Fouad YM Yehia R. Hepato-cardiac disorders. World J Hepatol. (2014) 6:41–54. 10.4254/wjh.v6.i1.41 24653793 PMC3953805

[61] Kopec M Duma A Helwani MA Brown J Brown F Gage BF Improving prediction of postoperative myocardial infarction with high-sensitivity cardiac troponin T and NT-proBNP. Anesth Analg. (2017) 124:398–405. 10.1213/ANE.0000000000001736 28002165 PMC5243152

[62] Loria P Marchesini G Nascimbeni F Ballestri S Maurantonio M Carubbi F Cardiovascular risk, lipidemic phenotype and steatosis. A comparative analysis of cirrhotic and non-cirrhotic liver disease due to varying etiology. Atherosclerosis. (2014) 232:99–109. 10.1016/j.atherosclerosis.2013.10.030 24401223

[63] Shi LY Jin R Lin CJ Wu JS Chen XW Yu Z B-type natriuretic peptide and cirrhosis progression. Genet Mol Res. (2015) 14:5188–96. 10.4238/2015.May.18.9 26125712

[64] Carl DE Sanyal A. The management of hepatorenal syndrome. Minerva Gastroenterol Dietol. (2009) 55:207–26.. 19305378

[65] Bruns T Zimmermann HW Stallmach A. Risk factors and outcome of bacterial infections in cirrhosis. World J Gastroenterol. (2014) 20:2542–54. 10.3748/wjg.v20.i10.2542 24627590 PMC3949263

[66] Lopez-Delgado JC Esteve F Manez R Torrado H Carrio ML Rodríguez-Castro D The influence of body mass index on outcomes in patients undergoing cardiac surgery: does the obesity paradox really exist? PLoS One. (2015) 10:e0118858. 10.1371/journal.pone.0118858 25781994 PMC4363511

[67] Kalaitzakis E. Gastrointestinal dysfunction in liver cirrhosis. World J Gastroenterol. (2014) 20:14686–95. 10.3748/wjg.v20.i40.14686 25356031 PMC4209534

[68] Rapp-Kesek D Ståhle E Karlsson TT. Body mass index and albumin in the preoperative evaluation of cardiac surgery patients. Clin Nutr. (2004) 23:1398–404. 10.1016/j.clnu.2004.06.006 15556262

[69] Jiménez Jiménez FJ Cervera Montes M Blesa Malpica AL. Metabolism and nutrition working group of the spanish society of intensive care medicine and coronary units. Guidelines for specialized nutritional and metabolic support in the critically-ill patient: update. Consensus SEMICYUC-SENPE: cardiac patient. Nutr Hosp. (2011) 26:76–80. 10.1590/S0212-16112011000800017 22411526

[70] Testani JM Khera AV St John Sutton MG Keane MG Wiegers SE Shannon RP Effect of right ventricular function and venous congestion on cardiorenal interactions during the treatment of decompensated heart failure. Am J Cardiol. (2010) 105:511–6. 10.1016/j.amjcard.2009.10.020 20152246 PMC2995805

[71] Diaz GC Moitra V Sladen RN. Hepatic and renal protection during cardiac surgery. Anesthesiol Clin. (2008) 26:565–90. 10.1016/j.anclin.2008.05.001 18765223

[72] Hemprich U Papadakos PJ Lachmann B. Respiratory failure and hypoxemia in the cirrhotic patient including hepatopulmonary syndrome. Curr Opin Anaesthesiol. (2010) 23:133–8. 10.1097/ACO.0b013e328335f024 20019600

[73] Peck-Radosavljevic M. Thrombocytopenia in chronic liver disease. Liver Int. (2017) 37:778–93. 10.1111/liv.13317 27860293

[74] Bezinover D Iskandarani K Chinchilli V McQuillan P Saner F Kadry Z Autoimmune conditions are associated with perioperative thrombotic complications in liver transplant recipients: a UNOS database analysis. BMC Anesthesiol. (2016) 16:26. 10.1186/s12871-016-0192-3 27207434 PMC4875607

[75] Irvine KM Ratnasekera I Powell EE Hume DA. Causes and consequences of innate immune dysfunction in cirrhosis. Front Immunol. (2019) 10:293. 10.3389/fimmu.2019.00293 30873165 PMC6401613

[76] Rueschenbaum S Ciesek S Queck A Widera M Schwarzkopf K Brüne B Dysregulated adaptive immunity is an early event in liver cirrhosis preceding acute-on-chronic liver failure. Front Immunol. (2021) 11:534731. 10.3389/fimmu.2020.534731 33574809 PMC7870861

[77] Rogers MA Blumberg N Saint S Langa KM Nallamothu BK. Hospital variation in transfusion and infection after cardiac surgery: a cohort study. BMC Med. (2009) 7:37. 10.1186/1741-7015-7-37 19646221 PMC2727532

[78] Trof RJ Danad I Groeneveld AJ. Global end-diastolic volume increases to maintain fluid responsiveness in sepsis-induced systolic dysfunction. BMC Anesthesiol. (2013) 13:12. 10.1186/1471-2253-13-12 23799933 PMC3698117

[79] Thielmann M Massoudy P Neuhäuser M Tsagakis K Marggraf G Kamler M Prognostic value of preoperative cardiac troponin I in patients undergoing emergency coronary artery bypass surgery with non-ST-elevation or ST-elevation acute coronary syndromes. Circulation. (2006) 114:I448–453. 10.1161/CIRCULATIONAHA.105.001057 16820617

[80] Madhwal S Atreja A Albeldawi M Lopez R Post A Costa MA. Is liver transplantation a risk factor for cardiovascular disease? A meta-analysis of observational studies. Liver Transpl. (2012) 18:1140–6. 10.1002/lt.23508 22821899

[81] Safadi A Homsi M Maskoun W Lane KA Singh I Sawada SG Perioperative risk predictors of cardiac outcomes in patients undergoing liver transplantation surgery. Circulation. (2009) 120:1189–94. 10.1161/CIRCULATIONAHA.108.847178 19752326

[82] Kim JH Lerose CC Landoni G Di Prima AL Licheri M Oriani A. Differences in biomarkers pattern between severe isolated right and left ventricular dysfunction after cardiac surgery. *J Cardiothorac Vasc Anesth*. (2020) 34:650–8. 10.1053/j.jvca.2019.07.128 31473115

[83] Bagshaw SM Bellomo R. The influence of volume management on outcome. Curr Opin Crit Care. (2007) 13:541–8. 10.1097/MCC.0b013e3282e2a978 17762233

[84] Alqahtani F Aljohani S Ghabra A Alahdab F Kawsara A Holmes DR Outcomes of transcatheter versus surgical aortic valve implantation for aortic stenosis in patients with hepatic cirrhosis. Am J Cardiol. (2017) 120:1193–7. 10.1016/j.amjcard.2017.06.067 28803656

[85] Choi YS Shim JK Hong SW Kim JC Kwak YL. Comparing the effects of 5% albumin and 6% hydroxyethyl starch 130/0.4 on coagulation and inflammatory response when used as priming solutions for cardiopulmonary bypass. Minerva Anestesiol. (2010) 76:584–91. 20661198

[86] Fernández Guerrero ML González López J Górgolas M. Infectious endocarditis in patients with cirrhosis of the liver: a model of infection in the frail patient. Eur J Clin Microbiol Infect Dis. (2010) 29:1271–5. 10.1007/s10096-010-0998-8 20549527

