# Supplementary material for: The importance of liver function assessment before cardiac surgery: A narrative review
Source: Front Surg. 2022 Dec 6;9:1053019. doi: 10.3389/fsurg.2022.1053019 (PMC9764862; doi:10.3389/fsurg.2022.1053019)
Supplement: Supplementary file 1 [file Table1.docx]

**Supplementary material:**

**Table S1: *Narrative review checklist*.**

| **Section/topic** | **Checklist item** | **Reported** |
| --- | --- | --- |
| ***Title*** | | |
| 1. Identify the report as a Narrative Review | | The present review has been identified as narrative review within the manuscript. |
| ***Abstract*** | | |
| 2. Provide an unstructured summary including, as applicable: background, objective, brief summary of narrative review and implications for future research, and clinical practice or policy development | | Unstructured abstract includes a rationale for the present narrative review together with the implications that liver disease entails for the patients. The abstract summarizes the importance of the narrative review. |
| ***Introduction****: Rationale/background* | | |
| 3. Describe the rationale for the review in the context of what is already known | | A strong rationale has been provided for the present review based on the most recent literature. |
| ***Objectives*** | | |
| 4. Specify the key question(s) identified for the review topic | | The aim of the present narrative review has been briefly described in introduction section. Most importantly, we have also described what is not reviewed (e.g., liver-related complications). |
| ***Methods****: Research selection* | | |
| 5. Specify the process for identifying the literature search (e.g., years considered, language, publication status, study design, and databases of coverage) when authors consider appropriate | | The process for identifying the literature search is not provided since this is not a systematic review. We have reviewed related literature reported within the last 20 years, but we have not provided the exact process since this is optional and it does not add significant information to the readers. |
| ***Discussion****: Narrative* | | |
| 1) Research reviewed including fundamental or key findings  2) Limitations and/or quality of the reviewed research  3) Need for future research | | We have discussed all important issues related with the topic of the present narrative review within the several subheadings.  Despite limitations and quality of the reviewed research is not specifically reported, the strength of recommendations/ suggestions is based on the nature of the reviewed literature (e.g., we wrote “we may suggest…” if the suggestion is based on observational studies).  Finally, we would like to underline that the need for future research in the topic is implicit and obvious along the whole manuscript. |
| ***Discussion****: Summary* | | |
| Provide an overall interpretation of the narrative review in the context of clinical practice | | We have provided different recommendations and suggestions for clinical practice within the manuscript based on the careful interpretation of the most recent and available literature |
